# Evaluation of tramadol human pharmacokinetics and safety after co-administration of magnesium ions in randomized, single- and multiple-dose studies

**DOI:** 10.1007/s43440-021-00239-x

**Published:** 2021-03-08

**Authors:** Piotr J. Rudzki, Katarzyna Jarus-Dziedzic, Monika Filist, Edyta Gilant, Katarzyna Buś-Kwaśnik, Andrzej Leś, Małgorzata Sasinowska-Motyl, Łukasz Nagraba, Magdalena Bujalska-Zadrożny

**Affiliations:** 1 Łukasiewicz Research Network - Pharmaceutical Research Institute, ul. Rydygiera 8, 02-091 Warsaw, Poland; 2 BioVirtus Research Site Sp, ul. Borowa 14/18, 05-400 Otwock, Poland; 3 Department of Pharmacodynamics, Centre for Preclinical Research and Technology, Medical University of Warsaw, ul. Banacha 1b, 01-793 Warsaw, Poland; 4 Orthopedic and Rehabilitation Department, Medical University of Warsaw, ul. Kondratowicza 8, 03-242 Warsaw, Poland

**Keywords:** Tramadol, O-Desmethyltramadol, Opioid analgesia, Pain treatment, Drug interaction, Pharmacokinetics

## Abstract

**Background:**

Magnesium ions (Mg^2+^) increase and prolong opioid analgesia in chronic and acute pain. The nature of this synergistic analgesic interaction has not yet been explained. Our aim was to investigate whether Mg^2+^ alter tramadol pharmacokinetics. Our secondary goal was to assess the safety of the combination.

**Methods:**

Tramadol was administered to healthy Caucasian subjects with and without Mg^2+^ as (1) single 100-mg and (2) multiple 50-mg oral doses. Mg^2+^ was administered orally at doses of 150 mg and 75 mg per tramadol dosing in a single- and multiple-dose study, respectively. Both studies were randomized, open label, laboratory-blinded, two-period, two-treatment, crossover trials. The plasma concentrations of tramadol and its active metabolite, O-desmethyltramadol, were measured.

**Results:**

A total of 25 and 26 subjects completed the single- and multiple-dose study, respectively. Both primary and secondary pharmacokinetic parameters were similar. The 90% confidence intervals for C_max_ and AUC_0-t_ geometric mean ratios for tramadol were 91.95–102.40% and 93.22–102.76%. The 90% confidence intervals for C_max,ss_ and AUC_0-τ_ geometric mean ratios for tramadol were 93.85–103.31% and 99.04–105.27%. The 90% confidence intervals for primary pharmacokinetic parameters were within the acceptance range. ANOVA did not show any statistically significant contribution of the formulation factor (p > 0.05) in either study. Adverse events and clinical safety were similar in the presence and absence of Mg^2+^.

**Conclusions:**

The absence of Mg^2+^ interaction with tramadol pharmacokinetics and safety suggests that this combination may be used in the clinical practice for the pharmacotherapy of pain.

**Graphic abstract:**

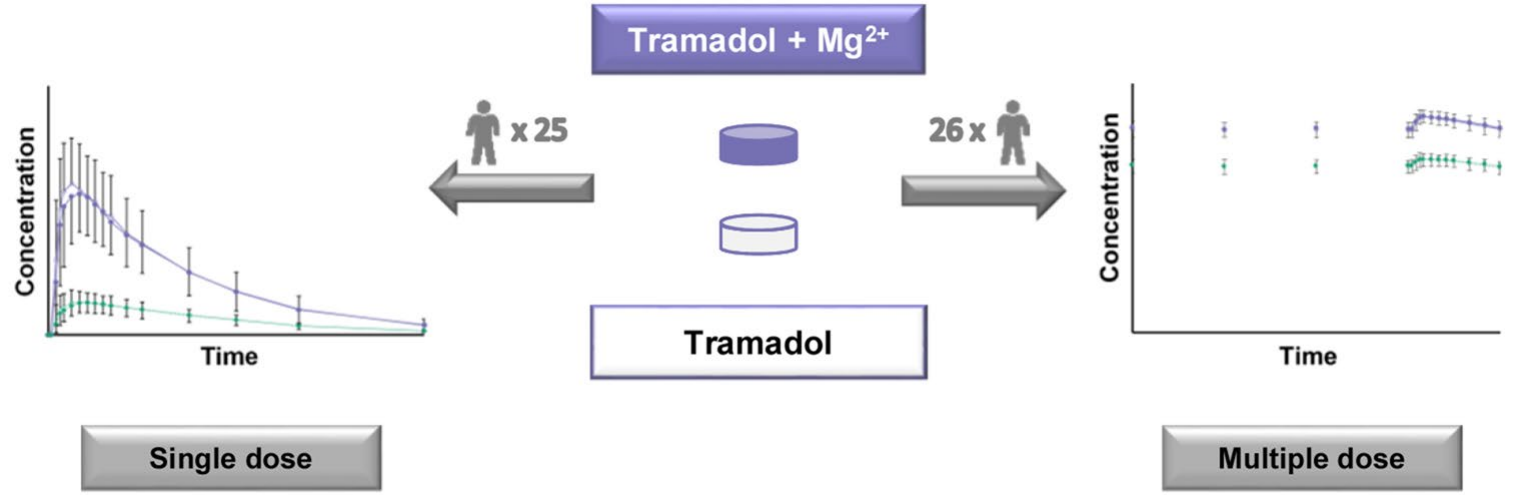

**Supplementary Information:**

The online version contains supplementary material available at 10.1007/s43440-021-00239-x.

## Introduction

Pain, especially chronic, decreases the quality of life and results in economic burdens for both individuals and the society in general [1]. Thus, it is important to continue research efforts for an effective pharmacotherapy of pain [2]. Opioids are still among the most powerful painkillers. Unfortunately, the use of these compounds is burdened with many side effects, such as respiratory depression, opioid hyperalgesia, development of tolerance, constipation, and addiction risk, which limits their use. In fact, despite a number of opioid prescription restrictions imposed several decades ago, we have witnessed opioid addiction that has reached epidemic proportions in the United States in the twentieth and twenty-first centuries. To reduce opioid doses (so-called sparing dose), and thus, reduce the risk of side effects, attempts have been made to combine opioids with adjuvant analgesics, such as anticonvulsants (gabapentin), antidepressants (amitriptyline), and N-methyl-d-aspartate (NMDA) receptor antagonists (ketamine). It is worth noting that magnesium ions [Mg^2+^] belong to the class of physiological antagonists of NMDA receptors [3, 4]. Results of own studies and the literature data demonstrated that Mg^2+^ increased opioid analgesia in experimental animal models of neuropathic [5–7], inflammatory [5], as well as acute [8–10] pain. Furthermore, the results of several clinical trials confirmed that parenterally administered Mg^2+^ reduces opioid consumption and improves postoperative pain scores without increasing opioid side effects [11–14]. Thus, the co-administration of opioids and Mg^2+^ may represent a valuable therapeutic option [15], but more data on the efficacy and safety of this combination are needed. Moreover, oral administration of opioids combined with magnesium salts would extend the use of this combination therapy in outpatient care.

The nature of synergistic analgesic interactions between opioids and Mg^2+^ remains unclear. However, it should be emphasize that Ionotropic NMDA receptors have been implicated in the underlying mechanisms of pain. The activation of these receptors removes the Mg^2+^ block in the channel. This results in calcium entering the cell and neuronal sensitization [16]. It was suggested that NMDA and opioid receptors are co-localized on the cell membrane of the same neuron and their C-termini are associated [17]. Moreover, the morphine-induced activation of the mu-opioid receptor leads to the phosphorylation of the Ser890 residue of the NMDA receptor’s C-terminus. This causes a dissociation of both receptors, an activation of the NMDA receptor, and an induction of further mechanisms leading to mu receptor phosphorylation and receptor desensitization [17, 18]. Thus, it can be assumed that Mg^2+^ reduces NMDA receptor-induced processes, decreases opioid receptor phosphorylation, and consequently enhances opioid analgesia [15].

Another hypothesis on the mechanism of Mg^2+^-potentiating opioid analgesia is the influence on opioid pharmacokinetics. The absorption process seems most likely to be affected, but increased Mg^2+^ content in the body may also influence distribution or metabolism, e.g., by changing conformation of proteins. Not all processes involved in tramadol distribution or metabolism are known and described. Thus, before defining the mechanism of hypothetical pharmacokinetic interaction, the first question is whether such an interaction exists. Our work focuses on one of the group of opioid painkiller—tramadol. The clinical pharmacology of this compound was reviewed by Grond and Sablotzky [19] and by Leppert [20]. After oral administration, the drug is rapidly and almost completely absorbed. A different enantiomeric ratio for tramadol and its main metabolites has been observed after intravenous and oral administration [21] and some gender differences have also been reported [22]. The mean plasma elimination half-life in healthy subjects after a single-dose administration ranges from 4.7 to 7.9 h [23–25]. After multiple dosing (50 mg every 6 h), the plasma steady state is reached within 3 days [26]. The influence of different pharmaceutical forms on the pharmacokinetics was investigated in elderly patients [27] and in patients who underwent total gastric resection [28]. Although the pharmacokinetics of tramadol and O-desmethyltramadol in humans is well described, we have failed to find any report describing how the pharmacokinetics may be altered by the co-administration of Mg^2+^.

Therefore, the aim of this study was to investigate whether the increased and prolonged tramadol analgesia in the presence of Mg^2+^ has any pharmacokinetic background. For this purpose, we have measured the concentrations of tramadol and its active metabolite, O-desmethyltramadol, after single and multiple oral administrations to healthy volunteers. The secondary goal of our study was to assess the safety of this combination.

## Methods

### Ethics and study design

The clinical study protocols were approved by an Independent Ethics Committee of the Warsaw Medical Chamber, Warsaw, Poland. Each subject signed a subject information and informed consent form prior to the screening procedures performed in accordance with the most recent version of the Declaration of Helsinki, current Good Clinical Practice guidelines, Polish laws governing the conduct of clinical investigations, and standard operating procedures of the clinical site. The studies were approved by the Polish Ministry of Health, registered by the Polish Regulatory Authorities – Central Register of Clinical Trials. The EudraCT numbers were 2014-004716-11 and 2014-004717-82 for the single- and multiple-dose studies, respectively.

Similar procedures were applied in both single- and multiple-dose studies, thus, the “Methods” section is combined. Both comparative pharmacokinetic studies were designed according to the European Medicines Agency (EMA) guideline on bioequivalence [29] as a randomized, open label, two-period, two-treatment, crossover trial. These single-site studies were conducted by BioVirtus Research Site Sp. z o.o. in Phase 1 Unit in Kajetany, Poland. Block randomization with block size of four was applied. The studies were laboratory-blinded with a wash-out period from 7 to 14 days. The same standard procedures were applied in both study periods, schemes for both studies are presented in the Supplementary Information section (Supplementary Figs. S1 and S2).

### Subjects

The following assumptions were adopted for the single-dose study: (1) within subject variation for C_max_ of no more than 20% [26], (2) ratio of pharmacokinetic parameters of no more than 1.05, and (3) a priori power of 0.80. The calculated sample size of 24 to complete the study was increased to 26 subjects to be randomized to account for the possible dropout. A total of 38 subjects were screened to meet this goal.

The following assumptions were made for the multiple-dose study: (1) within subject variation for C_max,ss_ of no more than 15% [26], (2) ratio of pharmacokinetic parameters of no more than 1.10, and (3) a priori power of 0.80. The calculated sample size of 22 to complete the study was increased to 30 subjects to be randomized to account for the possible dropout. A total of 56 subjects were screened to meet this goal.

The subjects enrolled met the following inclusion criteria: males and non-pregnant, non-lactating females 18–55 years old, body mass index (BMI) in the range of 18.5–30.0 kg/m^2^, non-smoker for at least 3 months before screening, and good general health condition assessed by medical history showing no co-morbidities, physical examination, normal electrocardiogram (ECG), and standard clinical laboratory tests within normal values.

Subjects were excluded if they met any of the exclusion criteria: history of clinically significant medical condition including arrhythmias and cardiac disease, hematologic condition and coagulation disorders, immunologic conditions, diseases of the musculoskeletal system, significant lung disease (bronchospastic respiratory disease), diabetes mellitus, kidney or liver insufficiency, endocrine disorders, neurologic or psychiatric disease, and active infection.

Subjects were also excluded in the following cases: drug abuse within last 5 years or alcohol abuse within 1 year, hypersensitivity or confirmed allergy to tramadol and/or to any excipient of the study products, history of gastrointestinal dysfunction, clinically significant abnormal or uncontrolled values of hematology, clinical chemistry or urinalysis that would affect the interpretation of the study data or the subject’s participation in the study, use of non-prescription drugs (including vitamins, herbal and dietary supplements) within 7 days or a prescription drug (excluding contraceptive pills and hormone replacement therapy) within 14 days or 5 half-lives (whichever longer) prior to the first dose of the study medication, or if the subject’s diet was deemed non-compliant by the investigator because of the inappropriate content of protein, fat and carbohydrates, or donation of at least 400 mL of blood within 90 days before the commencement of the study.

### Study products

The same products were used in both studies. The test product—50 mg *rac*-tramadol hydrochloride tablets with magnesium lactate corresponding to 75 mg of Mg^2+^—was manufactured by PozLab Sp. z o.o. (batch No. F03005, expiry date 09.2016). The reference product—without Mg^2+^—commercially available Tramadol Vitabalans^®^ 50-mg tablets was manufactured by Vitabalans Oy (batch No. 1197502, expiry date 12.2017).

### Study product administration

In both studies, the study product was administered orally with 250 mL of water at room temperature in the sitting position to be swallowed completely. Each subject’s oral cavity was checked following drug administration to confirm swallowing of the tablet. No drinking (except the water used for the product administration) was allowed from 1 h before to 1 h after the drug administration.

In the single-dose study, two 50-mg tablets of the test product or the reference product were administered in a fasted state on day 0 of each period. No food was allowed until 4 h after drug administration. In the multiple-dose study, one 50-mg tablet of the test product or the reference product was administered every 6 h on days 1 (two doses), 2, 3, 4, 5 and 6 (two doses) of each period. The total dose of tramadol in each period was 1000 mg. Subjects were fasted on day 1 for at least 4 h before the first dose and for 10 h before and 4 h after the last dose administration on day 6.

### Blood sampling

In both studies, the subjects had an intravenous cannula since day 0. Blood samples were centrifuged at 1500×*g* at 4 °C for 10 min, immediately after collection. The plasma was frozen in polypropylene vials at no less than − 65 °C for storage until the analysis.

In the single-dose study, blood samples were collected at 18 time points: pre-dose, 0.17; 0.5; 0.75; 1; 1.5; 2; 2.5; 3; 3.5; 4; 5; 6; 9; 12; 16; 24, and 36 h post-dose. In the multiple-dose study, blood samples were collected at 16 time points: 0 (pre-dose), and at 96, 102, 108, 114, 114.25, 114.5, 114.75, 115, 115.5, 116, 116.5, 117, 118, 119 and 120 h after the first dose administration. In total, a maximum of 187 mL and 175 mL of blood was collected from a single subject throughout the duration of the single- and multiple-dose studies, respectively.

### Bioanalysis of tramadol and O-desmethyltramadol

Tramadol and O-desmethyltramadol plasma concentrations were determined using the achiral liquid chromatography–mass spectrometry. Citrate was used as an anticoagulant for clinical samples, calibration standards, and quality control samples. Tramadol-d6 and O-desmethyltramadol-d6 were used as internal standards. Liquid–liquid extraction with tert-butyl methyl ether and sodium hydroxide was used for sample preparation (adapted from Tao et al. [30] and Godoy et al., [31], see also Supplementary Information). Novel conditions of chromatographic separation were applied using Kinetex biphenyl column (150 × 3.0 mm, 2.6 μm; Phenomenex, Torrance, CA, USA) at 45 ºC. The mobile phase was a mixture of 0.1% aqueous acetic acid and methanol (4:6 v/v). Ions of tramadol, O-desmethyltramadol, tramadol-d6, and O-desmethyltramadol-d6 were monitored at *m/z* ratio of 264.0, 250.0, 270.0, and 256.0, respectively. The study was conducted in compliance with the principles of Good Laboratory Practice.

### Pharmacokinetic and statistical analysis

The primary endpoints of the single-dose study were the maximum plasma concentration (*C*_max_) and the measured area under the plasma concentration vs. time curve (AUC_0-t_) for tramadol. The area under the plasma concentration vs. time curve extrapolated to infinity (AUC_0-∞_), the time to reach maximum plasma concentration of the drug (t_max_), and the elimination half-life (*t*_1/2_) for tramadol were selected as secondary parameters, which also included * C*_max_, AUC_0-t_, AUC_0-∞_, * t*_1/2_, and * t*_max_ for O-desmethyltramadol.

The primary endpoints of the multiple-dose study were the maximum plasma concentration in steady state (*C*_max,ss_) and the area under the plasma concentration vs. time curve in a dosing interval (AUC_0-τ_) for tramadol. Steady-state parameters, namely the time to reach maximum plasma concentration of the drug (*t*_max,ss_), minimum plasma concentration (*C*_min,ss_), average concentration (*C*_av,ss_), and peak trough fluctuation (PTF) for tramadol were selected as secondary parameters, which also included * C*_max,ss_, AUC_0-τ_, * t*_max,ss_, * C*_min,ss_, * C*_av,ss_, and PTF for O-desmethyltramadol.

The pharmacokinetic parameters were determined using Phoenix^®^ WinNonlin™ version 6.4 (Pharsight Corp.). * C*_max_ and * t*_max_ were obtained directly from experimental data. The elimination rate constant (*k*) was estimated from 3 to 6 points by least square regression analysis. The * t*_1/2_ was calculated as ln(2)/*k*. The AUC_0-t_ was calculated by the trapezoidal rule up to the last measurable plasma concentration (*C*_t_). The AUC_0-∞_ was calculated as the sum of AUC_0-t_ and the extrapolated area (AUC_rest_ = * C*_t_/ k).

The pharmacokinetics at steady state was studied for the dosing interval at 114–120 h after the first drug administration in a given period. The values of * C*_max,ss_, * C*_min,ss_, and * t*_max,ss_ were obtained directly from experimental data. The AUC_0-τ_ values were calculated by the trapezoidal rule. * C*_av,ss_ was calculated as AUC_0-τ_/τ. PTF was calculated as (*C*_max,ss_−*C*_min,ss_)/*C*_av,ss_ and presented as a percent value. The linear regression approach was used to verify whether the steady state was attained.

The SAS System for Windows version 9.4 was used for statistical analysis. Normal distribution of the primary pharmacokinetic parameters was tested using the Shapiro–Wilk, Kolmogorov–Smirnov, Cramer–von Mises and Anderson–Darling procedures at α = 0.05 significance level. The primary and secondary parameters except * t*_max_ were logarithmically transformed and subjected to the analysis of variance (ANOVA). The 90% confidence intervals of the test over the reference product’s geometric mean ratio of the primary pharmacokinetic parameters (AUC_0-t_ and * C*_max_ for tramadol in the single-dose study and * C*_max,ss_ and AUC_0-τ_ for tramadol in the multiple-dose study) were constructed. Mg^2+^ was assumed to have no influence when 90% confidence intervals were within the range of 80.00–125.00% [29]. Such analysis is equivalent to two one-sided Student’s *t* tests proposed by Schuirmann [32] with the null hypothesis of bioinequivalence (level of significance α = 0.05).

### Safety assessment

In both the studies, clinical safety had been assessed since the first drug administration and until the end of the study including subjects that did not complete the study. Monitoring of adverse events was complemented by the evaluation of standard clinical laboratory parameters, physical examinations, vital signs, and 12-lead ECG. The adverse events were classified by the system organ class and the preferred term using the Medical Dictionary for Regulatory Activities version 15.1. The adverse event severity was assessed subjectively and classified by investigators according to the National Cancer Institute Common Terminology Criteria for Adverse Events (CTCAE) version 4.02. Information on the adverse events was obtained from medical observations, laboratory test analyses, and spontaneous subject reporting after product administration. For every adverse event, the following data were recorded: type, severity, onset and resolution date, concomitant medications, relationship to the investigational products or procedures, and actions taken.

## Results

### Study population

In the single-dose study, 26 subjects (Table 1) met the inclusion criteria, and 25 subjects completed the study (Supplementary Fig. S3). One subject was withdrawn due to the positive result of toxicology on day 1 of Period 2.

**Table 1:** Demographic data of included subjects

Variable	Single-dose study		Multiple-dose study	
	Males Mean ± S.D.	Females Mean ± S.D.	Males Mean ± S.D.	Females Mean ± S.D.
*n*	22	4	24	6
Age (years)	31.7 ± 11.1	28.5 ± 6.4	31.3 ± 10.9	34.3 ± 10.1
Weight (kg)	81.2 ± 9.4	60.1 ± 8.2	76.8 ± 10.1	69.0 ± 7.7
Height (cm)	179.4 ± 5.6	166.5 ± 2.4	179.0 ± 6.7	163.0 ± 8.1
BMI (kg/m^2^)	25.3 ± 3.1	21.6 ± 2.5	23.9 ± 2.3	26.1 ± 3.4

*BMI* body mass index, *S.D.* standard deviation

In the multiple-dose study, 30 subjects were enrolled (Table 1) and 26 of them completed the study (Supplementary Fig. S4). Three subjects discontinued the study during Period 1 due to adverse events. One subject was withdrawn at the beginning of Period 2 due to the positive toxicology test for opiates.

### Bioanalytical method validation

All validation tests recommended by the EMA guideline [33] met the acceptance criteria (Supplementary Table S1). Calibration curves constructed by plotting the analyte-to-internal standard peak area ratios against the nominal concentrations were linear within the range of 5.0–750.0 ng/mL for tramadol and 2.5–150.0 ng/mL for O-desmethyltramadol. To confirm the method selectivity, blank human plasma from six different sources (including hemolyzed and hyperlipidemic plasma) were analyzed. There were no peaks influencing the quantification near the retention times of tramadol, O-desmethyltramadol, and the internal standards. The chromatograms of blank plasma samples containing *N*-desmethyltramadol, ibuprofen, and paracetamol did not show any significant interferences at the retention times of tramadol, O-desmethyltramadol, and the internal standards. Intra-run (within 1 day) and inter-run (within 3 days) accuracy and precision for both tramadol and O-desmethyltramadol met the acceptance criteria (Supplementary Tables S2 and S3). The stability of both analytes was confirmed using 90% confidence intervals (Supplementary Table S4, [34]). Relative standard deviation of normalized matrix factor was below 3% for 6 studied plasma sources including hemolyzed and hyperlipidemic plasma.

Quality control samples analyzed during the study met the acceptance criteria in all sequences. The method’s reliability during the study was further confirmed by the incurred samples reanalysis (ISR). The acceptance criteria, i.e., reanalysis within ± 20% of the mean concentration [33], in the single-dose study were met for 84 out of 92 samples (91%) in the case of tramadol, and for 85 out of 92 samples (92%) in the case of O-desmethyltramadol. The acceptance criteria in the multiple-dose study were met for 84 out of 86 samples (98%) in the case of tramadol, and for 81 out of 86 samples (94%) in the case of O-desmethyltramadol. No trends were observed on the complementary plots: %difference versus mean concentration and cumulative ISR plot (Supplementary Fig. S5 and S6) [35].

### Pharmacokinetic and statistical analysis

**Fig. 1:**
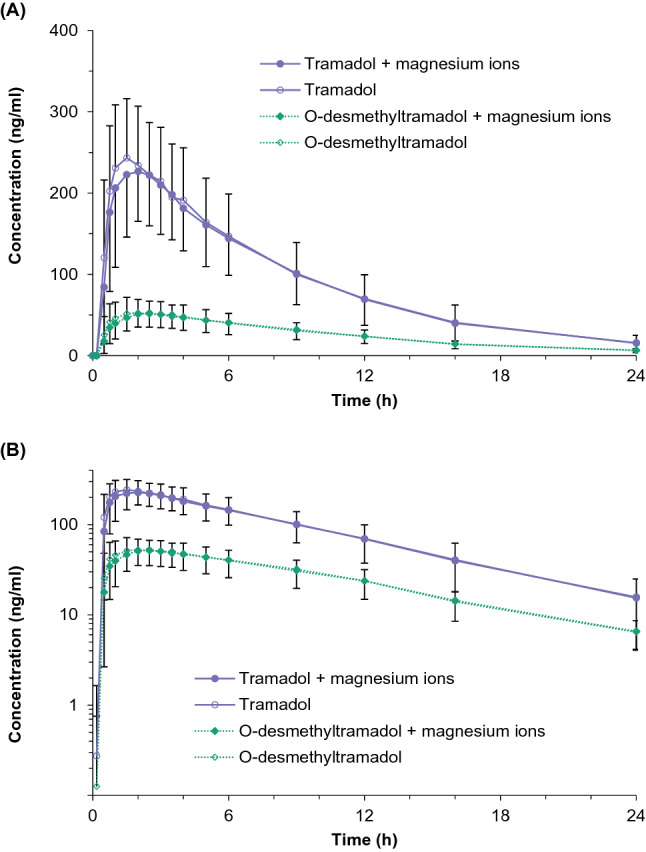
Mean tramadol (solid line) and O-desmethyltramadol (dotted line) plasma concentration vs. time curves in 25 healthy subjects following a single oral dose of tramadol (2 × 50 mg) with (filled points) and without magnesium ions (unfilled points) presented in the linear/linear scale (**A**) and log/linear scale (**B**)

**Fig. 2:**
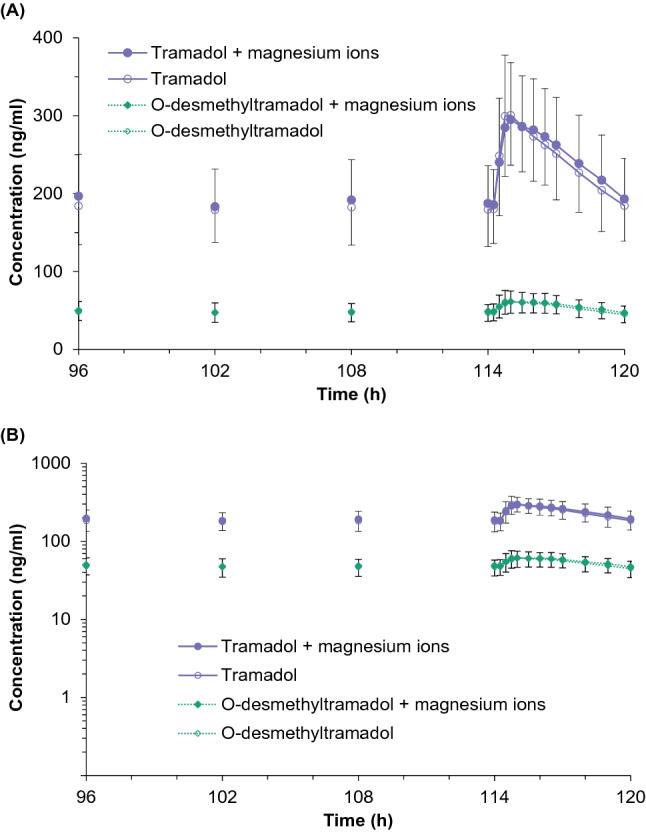
Mean tramadol (solid line) and O-desmethyltramadol (dotted line) plasma concentration vs. time curves in 26 healthy subjects following multiple oral doses of tramadol (50 mg) with (filled points) and without magnesium ions (unfilled points) presented in the linear/linear scale (**a**) and log/linear scale (**b**)

A hypothesis on the log-normal distribution of the primary parameters (*C*_max_, AUC_0-t_, * C*_max,ss_ and AUC_0-τ_) for tramadol for the test product and the reference product (*p* > 0.15) could not be rejected. The mean values of the primary and secondary pharmacokinetic parameters were similar for both formulations (Tables 2 and 3). The mean and individual plasma concentrations vs. time profiles in the presence and absence of Mg^2+^ were similar for both tramadol and O-desmethyltramadol (Figs. 1, 2 and S7–S14). A graphical presentation for the single-dose study was truncated at 24 h because only a few samples with the concentrations above the lower limit of quantification were recorded for both analytes at 36 h. In the single-dose study, the AUC_0-t_ to AUC_0-∞_ ratio was over 0.9 in all profiles for tramadol. As for O-desmethyltramadol, this ratio was higher than 0.8 in 47 out of 50 profiles (94%). In the multiple-dose study, the linear regression of the concentration vs. time was calculated at three time points (96, 102 and 108 h after the first dose administration in a given period). The slopes of the regression lines could not be differentiated from zero. Hence, the steady state was attained.

**Table 2:** Pharmacokinetic parameters for tramadol and O-desmethyltramadol after a single-dose administration of tramadol hydrochloride (2 × 50 mg) with and without Mg^2+^ to 25 healthy subjects

Parameter	Tramadol with Mg^2+^ mean ± S.D.	Tramadol without Mg^2+^ mean ± S.D.	Point estimate (90% confidence interval) (%)
*Tramadol*			
*C*_max_ (ng/ml)	264.7 ± 79.9	269.8 ± 70.3	97.03 (91.95–102.40)
AUC_0-t_ (ng^.^h/ml)	2167 ± 822	2222 ± 815	97.87 (93.22–102.76)
AUC_0-∞_ (ng^.^h/ml)	2265 ± 836	2318 ± 829	98.10 (93.31–103.13)
*t*_1/2_ (h)	5.49 ± 0.99	5.45 ± 0.97	100.71 (99.08–102.36)
*t*_max_ (h) ^a^	1.50 < 0.75–3.50 >	1.50 < 0.75–4.00 >	–
*O-Desmethyltramadol*			
*C*_max_ (ng/ml)	56.9 ± 18.1	58.2 ± 18.8	97.61 (91.84–103.76)
AUC_0-t_ (ng^.^h/ml)	624 ± 218	613 ± 183	100.25 (95.71–105.01)
AUC_0-∞_ (ng^.^h/ml)	673 ± 215	669 ± 181	99.12 (94.73–103.71)
*t*_1/2_ (h)	6.76 ± 1.24	6.66 ± 1.20	101.39 (99.67–103.15)
*t*_max_ (h) ^a^	2.50 < 0.75–3.50 >	2.00 < 0.75–4.00 >	–

*AUC*_*0-t*_ measured area under the plasma concentration vs. time curve, *AUC*_*0-∞*_ area under the plasma concentration vs. time curve extrapolated to the infinity, *C*_*max*_ maximum plasma concentration, *S.D.* standard deviation, *t*_*1/2*_ elimination half-life, *t*_*max*_ time to reach maximum plasma concentration

^a^Median, < minimum–maximum >

**Table 3:** Pharmacokinetic parameters for tramadol and O-desmethyltramadol after a multiple-dose administration of 50 mg tramadol hydrochloride with and without Mg^2+^ to 26 healthy subjects

Parameter	Tramadol with Mg^2+^ Mean ± S.D	Tramadol without Mg^2+^ Mean ± S.D	Point estimate (90% confidence interval) (%)
*Tramadol*			
*C*_max,ss_ (ng/mL)	315.1 ± 75.5	319.0 ± 73.8	98.47 (93.85–103.31)
AUC_0-τ_ (ng^.^h/mL)	1481 ± 360	1440 ± 314	102.11 (99.04–105.27)
*C*_min,ss_ (ng/mL)	177.7 ± 46.2	171.7 ± 43.8	103.14 (99.07–107.37)
*C*_av_ (ng/mL)	246.8 ± 59.9	240.1 ± 52.3	102.11 (99.04–105.27)
PTF (%)	56.4 ± 9.9	62.3 ± 18.8	92.84 (85.09–101.29)
*t*_max,ss_ (h) ^a^	115.00 < 114.50–117.00 >	115.00 < 114.50–116.50 >	–
*O-Desmethyltramadol*			
*C*_max,ss_ (ng/mL)	64.0 ± 14.0	64.4 ± 14.7	99.67 (96.11–103.35)
AUC_0-τ_ (ng^.^h/mL)	334 ± 78	324 ± 72	102.82 (100.04–105.68)
*C*_min,ss_ (ng/mL)	45.7 ± 12.1	43.3 ± 10.5	104.71 (101.06–108.49)
*C*_av_ (ng/mL)	55.6 ± 13.0	53.9 ± 11.9	102.82 (100.04–105.68)
PTF (%)	34.2 ± 8.9	39.4 ± 10.9	87.05 (78.74–96.23)
*t*_max,ss_ (h) ^a^	115.25 < 114.50–117.00 >	115.25 < 114.75–117.00 >	–

*AUC*_*0-τ*_ area under the plasma concentration vs. time curve in a dosing interval, *C*_*av*_ average concentration, *C*_*max,ss*_ maximum plasma concentration in a steady state, *C*_*min,ss*_ minimum plasma concentration in a steady state, *PTF* peak trough fluctuation, *S.D.* standard deviation, *t*_*max,ss*_ time to reach maximum plasma concentration

^a^Median, < minimum–maximum >

For the single-dose study, the 90% confidence intervals for both primary pharmacokinetic parameters (*C*_max_ and AUC_0-t_ for tramadol) were within the acceptance range of 80.00–125.00% (Table 2, estimated power > 0.999). The ANOVA indicated that the primary sources of variation for both tramadol and O-desmethyltramadol are attributed to the sequence and inter-subject effects, which were significant for all parameters (p ≤ 0.05, Table 4). The formulation effect, which corresponds to the Mg^2+^ influence, was not significant for the listed parameters (*p* > 0.05, Table 4).

**Table 4:** Results of the ANOVA (performed with the fixed effects model) of the logarithmically transformed pharmacokinetic parameters

Analyte	Parameter	Fixed effects/*p* value^†^			
		Sequence	Subject within sequence	Formulation	Period
*Single-dose study (n = 25)*					
	df	1	23	1	1
Tramadol	*C*_max_	**< 0.0001**	**< 0.0001**	0.4069	**0.0092**
	AUC_0-t_	**< 0.0001**	**< 0.0001**	0.4593	0.9411
O-Desmethyltramadol	*C*_max_	**< 0.0001**	**< 0.0001**	0.5080	0.8976
	AUC_0-t_	**< 0.0001**	**< 0.0001**	0.9363	0.7534
*Multiple-dose study (n = 26)*					
	df	1	24	1	1
Tramadol	*C*_max,ss_	0.2139	**< 0.0001**	0.5217	0.1996
	AUC_0-τ_	0.2118	**<0.0001**	0.3980	**0.0006**
O-Desmethyltramadol	*C*_max,ss_	**0.0337**	**<0.0001**	0.9584	0.1889
	AUC_0-τ_	**0.0009**	**<0.0001**	0.0851	0.3937

The statistically significant effects are presented in bold

*AUC*_*ss*_ area under the plasma concentration vs. time curve in a dosing interval, *C*_*max,ss*_ maximum plasma concentration in the steady state; *df* degrees of freedom

^†^Based on the type III sum of the squares and the least squares means

For the multiple-dose study, the 90% confidence intervals for both primary pharmacokinetic parameters (*C*_max,ss_ and AUC_0-τ_ for tramadol) were within the acceptance range of 80.00–125.00% (Table 3, estimated power > 0.999). The ANOVA indicated that the primary sources of variation for both tramadol and O-desmethyltramadol are attributed to the sequence and inter-subject effects that were significant for all parameters (*p* ≤ 0.05, Table 4). The formulation effect for the listed parameters was not significant (*p* > 0.05, Table 4).

### Safety

There were neither serious adverse events nor deaths recorded during both studies. All adverse events were mild to moderate in severity. There were no clinically significant changes in the mean clinical laboratory test results, electrocardiogram findings, or vital signs. The physical examination results remained unchanged for all the participants.

In the single-dose study, 20 adverse events were reported in 12 subjects (46%). Out of 26 subjects who received the study products in Period 1, 9 subjects (35%) experienced 12 adverse events; 5 adverse events were classified as possibly related and 7 as not related to the study products. After the product administration in Period 2, 6 subjects (23%) experienced a total of 8 adverse events; 4 adverse events were classified as possibly related and 4 as not related to the study products. The types of adverse events were similar across both the treatments (Table 5, Supplementary Table S5).

**Table 5:** Sum of adverse events (percentage) of subjects with adverse events)

Related to the study product	Tramadol with Mg^2+^	Tramadol without Mg^2+^
*Single-dose study*	*n* = 25	*n* = 26
Possibly	5 (20%)	4 (15%)
Not related	7 (28%)	4 (15%)
*Multiple-dose study*	*n* = 29	*n* = 27
Probably	12 (41%)	5 (19%)
Possibly	28 (97%)	25 (93%)
Unlikely	14 (48%)	8 (30%)
Not related	21 (71%)	23 (85%)

Number of subjects in a particular group during a single- and multiple-dose study refers to the number of treated subjects

In the multiple-dose study, 136 adverse events were reported in 26 subjects (87%). Out of 30 subjects who received the study products in Period 1, 26 subjects experienced 91 adverse events (14 adverse events were classified as probably related, 36 as possibly related, 15 as unlikely related, and 26 as not related to the study products). After the product administration in Period 2, 15 subjects out of 26 continuing the study experienced a total of 33 adverse events (1 adverse event was classified as probably related, 11 as possibly related, 6 as unlikely related, and 15 as not related to the study products). After being discharged from the clinic in Period 2 and before the follow-up visit, 10 subjects out of 26 continuing the study experienced a total of 12 adverse events (2 adverse events were classified as probably related, 6 as possibly related, 1 as unlikely related and, 3 as not related to the study products). The types of adverse events were similar across both treatments (Table 5, Supplementary Table S6).

## Discussion

The results of the study indicate that Mg^2+^ ions do not alter tramadol pharmacokinetics. Both primary and secondary pharmacokinetic parameters were similar, with overlapping average plasma profiles. The 90% confidence intervals for the geometric mean ratios of the primary pharmacokinetic parameters met the acceptance criteria, and the formulation factor in ANOVA was not significant. The adverse events were also similar in the subjects receiving tramadol with and without Mg^2+^.

Magnesium deficiency is a common condition in Poland and it may mask the influence of Mg^2+^. Therefore, a single-dose approach would not be sufficient to draw valid conclusions. However, it would be unethical and unnecessary to conduct a multiple-dose study if the single-dose study revealed a significant influence of Mg^2+^ on tramadol pharmacokinetics. Since Mg^2+^ effect was not observed in the single-dose study, a confirmatory study after a multiple-dose administration was conducted. The design of both studies enabled us to draw valid conclusions. The bioanalytical method had an appropriate range to measure tramadol and O-desmethyltramadol concentrations. Sampling schedules enabled proper characterization of the pharmacokinetic profiles. Tramadol and O-desmethyltramadol pharmacokinetics reported in this paper are in line with literature data for achiral methods [23, 24]. Pharmacokinetics in overweight subjects were similar to normal weight subjects, in line with observations of Porażka et al. [36]. The studies were conducted according to the EMA bioequivalence guideline [29] because its methodology is appropriate for comparing drug formulations with and without Mg^2+^.

The population consisting of healthy subjects may be considered a limitation of the study. Healthy subjects were selected to avoid any influence of co-morbidities and concomitant medications on tramadol pharmacokinetics. Hence, just as in bioequivalence studies, some differences in pharmacokinetics between patients and healthy subjects can be anticipated. In our study, we have observed very narrow confidence intervals and failed to find any indication that the interaction of Mg^2+^ with tramadol would be different in both the groups.

The CYP2D6 gene—responsible for the metabolism of tramadol to O-desmethyltramadol—is highly polymorphic. We decided to avoid genotyping and exclusion of poor and ultra-rapid CYP2D6 metabolizers to make the study population more representative for the general population. In the single-dose study, C_max_ for tramadol ranged from 132.51 to 458.16 ng/mL for the test product and from 155.54 to 435.22 ng/mL for the reference product. These values suggest differences in metabolism of tramadol in particular subjects. However, they did not influence the study results as 90% confidence intervals for geometric means test to reference ratio were within the limits for primary pharmacokinetic parameters. Still, this issue may be interesting for further research.

In our studies, Mg^2+^ was not defined as an active substance, thus, its determination was not required. We hope that this paper will inspire future research on Mg^2+^ levels monitoring in opioid-treated patients. In addition, one may argue that Mg^2+^ is present in numerous medicinal products containing opioids—including the reference product in these studies—as magnesium stearate. Both very low solubility and low content in tablets disqualify this excipient as a source of Mg^2+^ and preclude its interaction with opioids.

As expected, the frequency of adverse events was considerably higher in the multiple-dose study (87%) than in the single-dose study (46%). Typical adverse events for tramadol such as nausea and vomiting were observed and classified as probably related to the product. The results confirmed that the prophylaxis for these symptoms was not required in any subjects. There were no novel or atypical adverse event related to the study product observed (Table 5, Supplementary Tables S5 and S6).

The results of our studies do not support the hypothesis that the increased and prolonged analgesia of tramadol in the presence of Mg^2+^ is based on the pharmacokinetics of the parent drug and its main metabolite. Moreover, the analyses of the adverse events indicate that Mg^2+^ does not alter the safety of tramadol therapy. The absence of Mg^2+^ influence on the pharmacokinetics and adverse events of tramadol confirms the safety of this combination in clinical practice.

In a multicenter, single blinded, parallel-group study to assess efficacy, safety and tolerability of the combination of tramadol with magnesium lactate in the management of chronic pain in subjects with osteoarthritis of the hip or/and knee, it was demonstrated that the combined oral administration of magnesium salt with tramadol at a lower dose induced a comparable analgesic effect as a reference product—tramadol at a higher dose (data to be published in the future). Unfortunately, the increase of opioid analgesia after oral administration of Mg^2+^ has not been confirmed in cancer patients receiving morphine [37]. Due to these discrepancies, further clinical studies focused on other types of pain are needed; however, the results of our pharmacokinetic research suggest that the co-administration of opioids (particularly tramadol) with Mg^2+^ may be considered as a promising novel pharmacotherapy of pain. This research is particularly important in the light of the over-prescription of opioids in recent years, and thus, the spreading wave of opioid addiction worldwide. [4, 38–43]. The addition of Mg^2+^ may help to adequately control opioid treatment by reducing consumption, and thus, reducing adverse events [11–13].

## Conclusions

In both single- and multiple-dose studies, the absence of Mg^2+^ influence on tramadol pharmacokinetics and safety was observed after oral administration to healthy subjects. Pharmacokinetic parameters and profiles for tramadol and O-desmethyltramadol were similar. The lack of pharmacokinetic interaction between Mg^2+^ and tramadol suggests that the combination of tramadol and Mg^2+^ is safe in clinical practice, supporting the co-administration of opioids with Mg^2+^ as a promising novel strategy for the pharmacotherapy of pain.

## Supplementary Information

Below is the link to the electronic supplementary material.

Supplementary file1 (DOCX 1820 KB)
