# Supplementary material for: Evaluation of tramadol human pharmacokinetics and safety after co-administration of magnesium ions in randomized, single- and multiple-dose studies
Source: Pharmacol Rep. 2021 Mar 8;73(2):604–14. doi: 10.1007/s43440-021-00239-x (PMC7994227; doi:10.1007/s43440-021-00239-x)

**Supplementary Information to**

**Evaluation of tramadol human pharmacokinetics and safety
after co-administration of magnesium ions
in randomized, single- and multiple-dose studies**

**Pharmacological Reports**

**Correspondence:** Magdalena Bujalska-Zadrożny, PhD, Department of Pharmacodynamics, Centre for Preclinical Research and Technology , Medical University of Warsaw, 1b Banacha Street, 01-793 Warsaw, Poland.
*Tel.*: +48 22 116 61 26; *fax*: +48 22 116 62 03;

*e-mail*: [mbujalska@gmail.com](mailto:mbujalska@gmail.com); [magdalena.bujalska@wum.edu.pl](mailto:magdalena.bujalska@wum.edu.pl)

Piotr J. Rudzki^1^, Katarzyna Jarus-Dziedzic^2,*^, Monika Filist^1^, Edyta Gilant^1^, Katarzyna Buś-Kwaśnik^1^, Andrzej Leś^1^, Małgorzata Sasinowska-Motyl^3^, Łukasz Nagraba^4^, Magdalena Bujalska-Zadrożny^3^

^1^ Łukasiewicz Research Network - Pharmaceutical Research Institute, 8 Rydygiera Street, 02-091 Warsaw, Poland

^2^ BioVirtus Research Site Sp. z o.o., 14/18 Borowa Street, 05-400 Otwock, Poland

^3^ Department of Pharmacodynamics, Centre for Preclinical Research and Technology, Medical University of Warsaw, 1b Banacha Street, 01-793 Warsaw, Poland

^4^ Orthopedic and Rehabilitation Department, Medical University of Warsaw, Kondratowicza 8 Street, 03-242 Warsaw, Poland

^*^ Principal investigator, present address: BioResearch Group Sp. z o.o., 9/U-2 Sokołowska Street, 01-142 Warsaw, Poland

**Sample preparation procedure (adapted from Tao et al. [30] and Godoy et al, [31])**

- 250 μL of human plasma was vortex mixed with and 25 μL of internal standard working solution (2.50 μg/mL of Tramadol-d6 and 0.75 μg/mL of O-desmethyltramadol-d6)
- 20 μL of 1 M NaOH was added and vortex mixed
- 2 mL of methyl tert-butyl ether was added and vibrax mixed for 5 min (750 rpm)
- the sample was centrifuged for 5 minutes at 3500 rpm
- the aqueous phase was frozen and the organic phase was transferred to a clean test tube
- the organic solvent was evaporated under the stream of nitrogen
- the dry residue was dissolved in 150 μL of the mobile phase and vortex mixed
- the solution was transferred to a chromatographic vial

**Chromatographic conditions**

- Column: Kinetex Biphenyl 150 x 3.0 mm, 2.6 μm (Phenomenex, Torrance, CA, USA) connected to a guard column SecurityGuard C18, 4.0 x 3.0 mm or SecurityGuard
- ULTRA Cartridges UHPLC Biphenyl (Phenomenex, Torrance, CA, USA)
- Mobile phases: 0.1% (v/v) aqueous CH_3_COOH:MeOH 2:3 (v/v)
- Isocratic elution
- Flow rate: 0.25 mL/min
- Injection volume: 15 µL
- Oven temperature: 45 ± 2 ºC
- Run time: 7.5 min

**Instruments and chemicals**

The high-performance liquid chromatography system consisted of pump LC-10ADVP connected to an autosampler SIL-HTA, a degaser DGU-20A3, a column oven CTO-10A and a mass spectrometer LCMS-2010. The data processing software was LCMSsolution v. 2.05 (Shimadzu, Duisburg, Germany).

- Tramadol hydrochloride (reference standard), Seneca pharmaceuticals, **Hlohovec, Slovakia**
- O-desmethyltramadol hydrochloride (reference standard), LGC GmbH, Luckenwalde, Germany
- Tramadol-d6 hydrochloride (internal standard for Tramadol), TLC Pharmaceutical Standards, Vaughn, Canada
- O-desmethyltramadol-d6 hydrochloride (internal standard for O-desmethyltramadol), TLC Pharmaceutical Standards, Vaughn, Canada
- Methanol, MeOH, LC-MS grade, Avantor Performance Materials Poland, Gliwice, Poland
- *Tert*-butyl methyl ether, TBME, HPLC grade, S. Witko, Łódź, Poland
- Acetic acid, CH3COOH, ≥ 99.5%, pure p.a., Chempur, Piekary Śląskie, Poland
- Sodium hydroxide, NaOH, pure, Avantor Performance Materials Poland, Gliwice, Poland
- Water from Milli - Q system, Millipore

**Table S1**

Method validation summary

| **Validation parameter** | **Acceptance criteria** |
| --- | --- |
| Linearity:  TRM: 5.00-750.00 ng/mL  ODT: 2.50-150.00 ng/mL | - Accuracy for at least 6 out of 8 calibration standards within 85-115% (for LLOQ 80-120%) - Correlation coefficient of calibration curves ≥ 0.95 |
| Carry over | - Peak area of interfering compounds in blank plasma ≤ 20% of LLOQ for TRM and ODT as well as ≤ 5% of working concentrations for IS-T and IS-O |
| Selectivity  -blank plasma samples  -blank plasma with N-desmethyltramadol  - blank plasma samples with ibuprofen and paracetamol | - Peak area of interfering compounds ≤ 20% of LLOQ for TRM and ODT as well as ≤ 5% of working concentrations for IS-T and IS-O |
| Selectivity  - QC samples with N-desmethyltramadol | - 90% CI for accuracy should fall within 85-115% |
| Matrix effect | - The RSD of the IS-normalized Matrix Factor ≤ 15% |
| Extraction recovery  of TRM and ODT | - For each studied QC level the mean recovery should be consistent |
| Extraction recovery  of IS-T and IS-O | - Recovery of IS-T should not depend on TRM and ODT concentrations |
| Sensitivity/LLOQ  TRM: 5.00 ng/mL  ODT: 2.50 ng/mL | - Signal to noise ratio (S/N) ≥ 5 - The upper limit of 90% CI for within-run and between-run precision ≤ 20% - 90% CI for within-run and between-run accuracy within 80-120% |
| Accuracy and precision:  QC samples  TRM: 15.00,100.00, 250.00, 350.00 and 600.00 ng/mL  ODT: 7.50, 35.00, 55.00, 70.00 and 120.00 ng/mL | - The upper limit of 90% CI for within-run and between-run precision ≤ 15% - 90% CI for within-run and between-run accuracy within 85-115% |
| Dilution integrity | - The upper limit of 90% CI for precision ≤ 15% - 90% CI for accuracy within 85-115% |
| Stability of solutions:  stock solutions at room temp. and freezer  working solutions at room temp. and refrigerator | - 90% CI for the mean stability within 90-110% |
| Stability of analytes in plasma samples:  Freeze-thaw (3 cycles; deep freezer)  Short-term (4 h; room temp.)  Long-term (21 days; freezer)  Long-term (82 days; deep freezer) | - The mean concentrations of test and reference samples within ±15% of nominal concentration - 90% CI for mean stability within 85-115% |
| Autosampler stability (68 h; room temp.) | - The mean concentrations of test and reference samples within ±15% of nominal concentration - 90% CI for mean stability within 85-115% |

*CI* confidence interval; *IS-T* tramadol-d6; *IS-O* O-desmethyltramadol-d6; *LLOQ* lower limit of quantification; *ODT* O-desmethyltramadol; *QC* quality control; *TRAM* tramadol.

**Table S2**

The accuracy and precision of the tramadol determination in plasma samples (*n* = 6)

| **Nominal concentration** | **2.5 ng/mL** | **7.5 ng/mL** | **55.0 ng/mL** | **120.0 ng/mL** |
| --- | --- | --- | --- | --- |
| Intra-run | | | | |
| Mean ± SD (ng/mL) | 5.1 ± 0.2 | 14.6 ± 0.3 | 240.5 ± 5.7 | 613.5 ± 8.0 |
| Accuracy (90% CI, %) | 99.0-104.8 | 96.1-99.0 | 94.3-98.1 | 101.2-103.3 |
| Precision (90% CI, %) | 2.57-6.15 | 1.34-3.21 | 1.75-4.19 | 0.95-2.29 |
| Inter-run | | | | |
| Mean ± SD (ng/mL) | 5.0 ± 0.2 | 14.9 ± 0.5 | 253.9 ± 4.5 | 600.0 ± 10.6 |
| Accuracy (90% CI, %) | 98.8-102.5 | 98.2-101.1 | 100.8-102.3 | 99.3-100.7 |
| Precision (90% CI, %) | 3.69-5.78 | 3.01-4.71 | 1.47-2.31 | 1.46-2.29 |

*CI* confidence interval

**Table S3**

The accuracy and precision of the O-desmethyltramadol determination in plasma samples (*n* = 6)

| **Nominal concentration** | **2.5 ng/mL** | **7.5 ng/mL** | **55.0 ng/mL** | **120.0 ng/mL** |
| --- | --- | --- | --- | --- |
| Intra-run | | | | |
| Mean ± SD (ng/mL) | 2.5 ± 0.1 | 7.3 ± 0.6 | 53.5 ± 1.3 | 117.7 ± 2.0 |
| Accuracy (90% CI, %) | 96.7-106.0 | 90.9-103.1 | 95.3-99.2 | 96.7-99.5 |
| Precision (90% CI, %) | 4.11-9.85 | 5.62-13.45 | 1.80-4.31 | 1.27-3.04 |
| Inter-run | | | | |
| Mean ± SD (ng/mL) | 2.4 ± 0.2 | 7.2 ± 0.5 | 57.1 ± 3.7 | 121.8 ± 7.6 |
| Accuracy (90% CI, %) | 93.5-99.2 | 93.8-99.2 | 101.0-106.5 | 98.9-104.2 |
| Precision (90% CI, %) | 6.00-9.41 | 5.64-8.83 | 5.31-8.33 | 5.19-8.13 |

*CI* confidence interval

**Table S4**

Stability of analytes in plasma expressed as 90% confidence intervals (*n* = 6) [34]

| **Nominal concentration** | **15 ng/mL**  **TRM** | **600 ng/mL**  **TRM** | **7.5 ng/mL**  **ODT** | **120 ng/mL**  **ODT** |
| --- | --- | --- | --- | --- |
| Freeze-thaw, 3 cycles at ≤ -65 °C | 98.5-106.1% | 97.1-102.8% | 87.9-93.8% | 86.0-92.2% |
| Short-term, 4 hours at room temp. | 97.5-104.5% | 95.6-101.5% | 93.6-98.3% | 88.9-95.4% |
| Long-term, 82 days at ≤ -65 °C | 97.7-103.8% | 92.5-93.9% | 104.6-113.1% | 90.2-91.3% |

*ODT* O-desmethyltramadol; *TRM* tramadol

**Table S5**

Number (percentage) of subjects with adverse events in a single-dose study.

| Adverse event | Tramadol with Mg^2+^  (n = 25) | Tramadol without Mg^2+^  (n = 26) |
| --- | --- | --- |
| Possibly related to the study product | | |
| Sum of adverse events | 5 (20%) | 4 (15%) |
| Orthostatic fainting | 2 (8%) | 2 (8%) |
| Weakness | 2 (8%) | 0 (0%) |
| Malaise | 0 (0%) | 1 (4%) |
| Heart palpitations | 0 (0%) | 1 (4%) |
| Dizziness | 1 (4%) | 0 (0%) |
| Not related to the study product | | |
| Sum of adverse events | 7 (28%) | 4 (15%) |
| Common cold | 2 (8%) | 1 (4%) |
| Orthostatic hypotension | 0 (0%) | 1 (4%) |
| Increased blood pressure | 1 (4%) | 0 (0%) |
| Sore throat | 0 (0%) | 1 (4%) |
| Orthostatic fainting | 1 (4%) | 0 (0%) |
| Allergic rhinitis | 0 (0%) | 1 (4%) |
| Muscles pain | 1 (4%) | 0 (0%) |
| Elevated levels of alanine aminotransferase | 1 (4%) | 0 (0%) |
| Elevated levels of bilirubin | 1 (4%) | 0 (0%) |

**Table S6**

Number (percentage) of subjects with adverse events in a multiple-dose study.

| Adverse event | Tramadol with Mg^2+^  (n = 29) | Tramadol without Mg^2+^  (n = 27) |
| --- | --- | --- |
| Probably related to the study product | | |
| Sum of adverse events | 12 (41%) | 5 (19%) |
| Somnolence | 4 (14%) | 0 (0%) |
| Discontinuation syndrome | 1 (3%) | 1 (4%) |
| Difficulty in starting urination | 1 (3%) | 1 (4%) |
| Vomiting | 2 (7%) | 0 (0%) |
| Nausea | 1 (3%) | 1 (4%) |
| Dry mouth and nose | 0 (0%) | 1 (4%) |
| Dry mouth | 0 (0%) | 1 (4%) |
| Hyperhidrosis | 1 (3%) | 0 (0%) |
| Hands paresthesia | 1 (3%) | 0 (0%) |
| Syncope | 1 (3%) | 0 (0%) |
| Possibly related to the study product | | |
| Sum of adverse events | 28 (97%) | 25 (93%) |
| Somnolence | 4 (14%) | 8 (30%) |
| Dizziness | 3 (10%) | 8 (30%) |
| Vomiting | 3 (10%) | 2 (7%) |
| Difficulty in starting urination | 3 (10%) | 2 (7%) |
| Nausea | 3 (10%) | 1 (4%) |
| Headache | 5 (17%) | 0 (0%) |
| Abdominal pain | 0 (0%) | 1 (4%) |
| Insomnia | 1 (3%) | 0 (0%) |
| Hot flashes | 1 (3%) | 0 (0%) |
| Flatulence | 1 (3%) | 0 (0%) |
| Dry mouth | 1 (3%) | 0 (0%) |
| Constipation | 0 (0%) | 1 (4%) |
| Fast fatigue | 1 (3%) | 0 (0%) |
| Rash | 0 (0%) | 1 (4%) |
| Orthostatic fainting | 1 (3%) | 0 (0%) |
| Agitation | 0 (0%) | 1 (4%) |
| Attention deficit disorder | 1 (3%) | 0 (0%) |
| Unlikely related to the study product | | |
| Sum of adverse events | 14 (48%) | 8 (30%) |
| Headache | 4 (14%) | 1 (4%) |
| Nausea | 2 (7%) | 1 (4%) |
| Dizziness | 2 (7%) | 1 (4%) |
| Weakness | 3 (10%) | 0 (0%) |
| Somnolence | 0 (0%) | 2 (7%) |
| Itching | 1 (3%) | 0 (0%) |
| Rash | 0 (0%) | 1 (4%) |
| Conjunctivitis | 1 (3%) | 0 (0%) |
| Sensation of hot | 0 (0%) | 1 (4%) |
| Chest pain on the right side | 0 (0%) | 1 (4%) |
| Difficult in defecation | 1 (3%) | 0 (0%) |
| Not related to the study product | | |
| Sum of adverse events | 21 (71%) | 23 (85%) |
| Headache | 6 (20%) | 9 (33%) |
| Nausea | 3 (10%) | 4 (15%) |
| Constipation | 3 (10%) | 1 (4%) |
| Dizziness | 2 (7%) | 1 (4%) |
| Vomiting | 1 (3%) | 2 (7%) |
| Lower back pain | 2 (7%) | 0 (0%) |
| Toothache | 2 (7%) | 0 (0%) |
| Common cold | 0 (0%) | 1 (4%) |
| Sore throat | 0 (0%) | 1 (4%) |
| Orthostatic fainting | 0 (0%) | 1 (4%) |
| Allergic rhinitis | 0 (0%) | 1 (4%) |
| Herpes | 1 (3%) | 0 (0%) |
| Weakness | 0 (0%) | 1 (4%) |
| Right hip pain | 1 (3%) | 0 (0%) |
| Pain of left hypochondriac region | 0 (0%) | 1 (4%) |

**Figure S1** Study scheme for a single-dose study


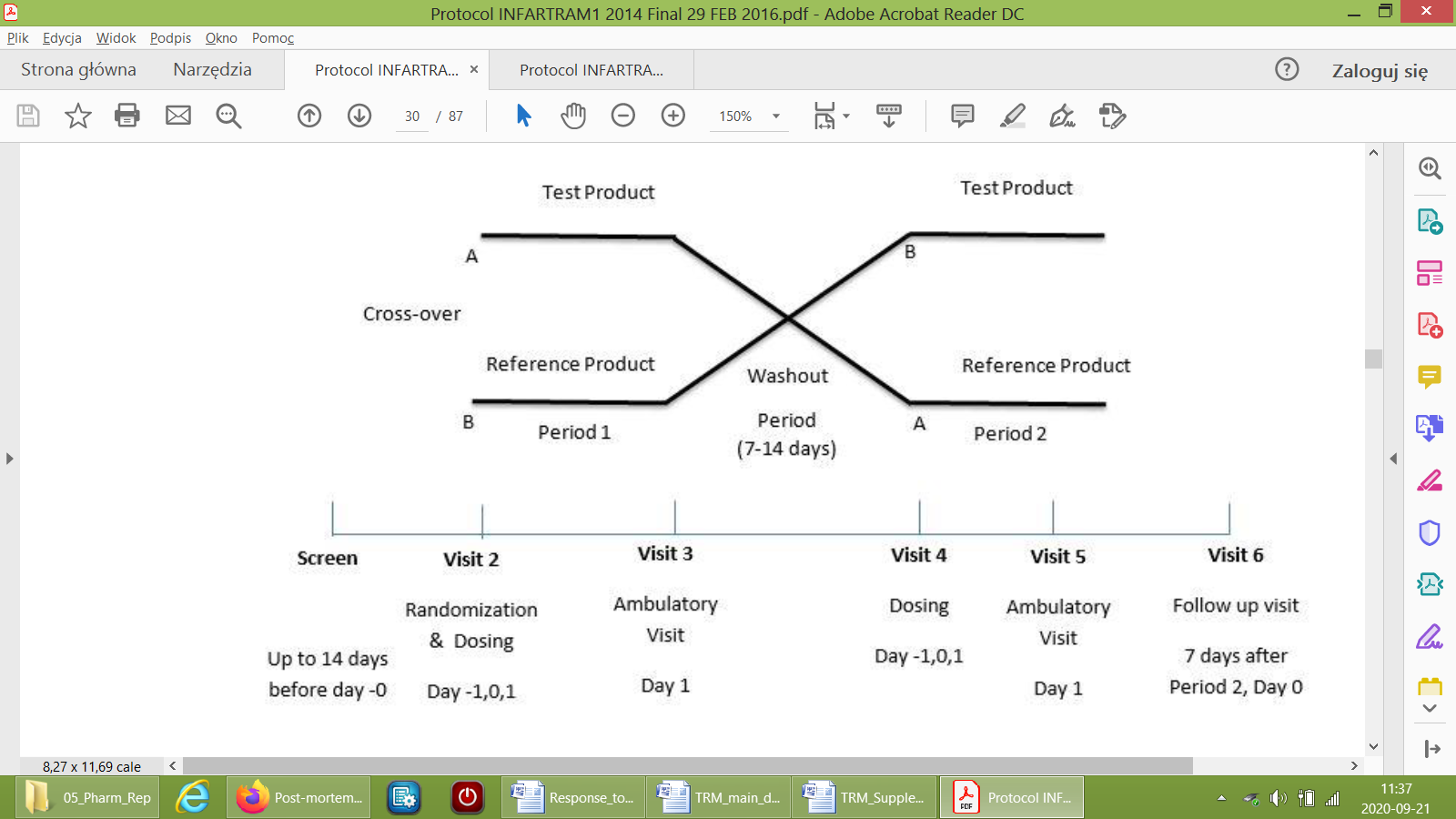


**Figure S2** Study scheme for a multiple-dose study


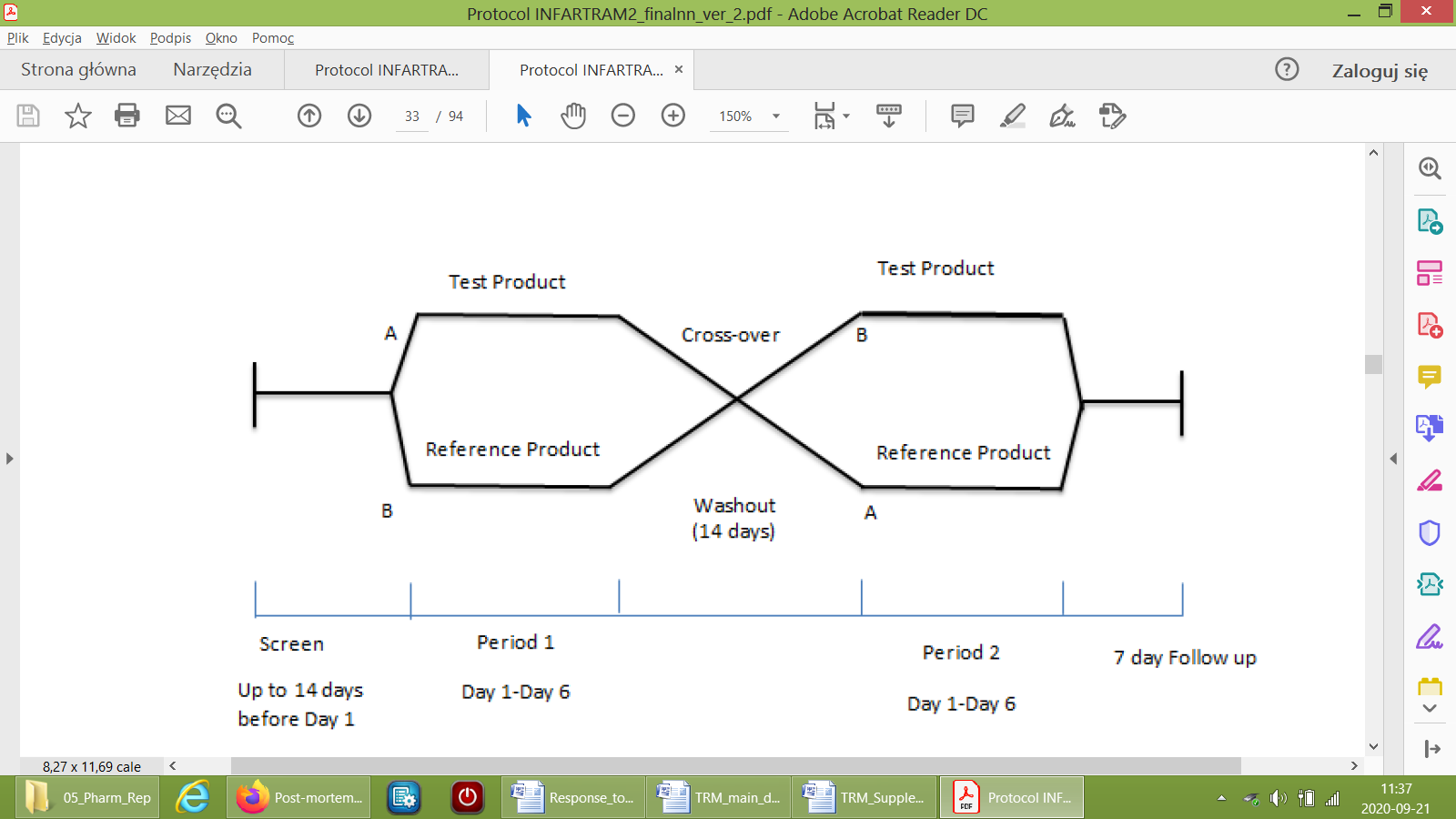


**Figure S3** Flow chart for a single-dose study


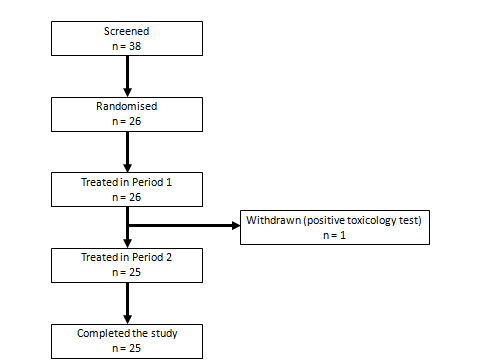


**Figure S4** Flow chart for a multiple-dose study


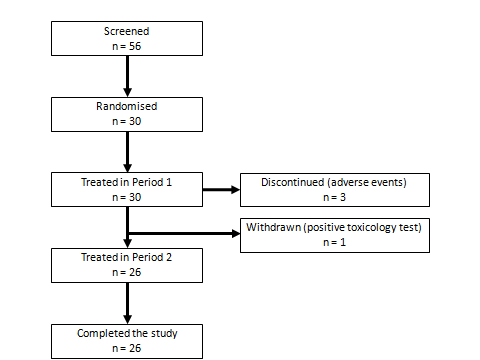


**Figure S5** Visual presentation of the incurred samples reanalysis (ISR) according to Rudzki et al. [35] in the single-dose study for tramadol (violet symbols;
A – %difference versus mean concentration; B – cumulative ISR plot)
and O-desmethyltramadol (green symbols; C – %difference versus mean concentration; D – cumulative ISR plot).

| (A)  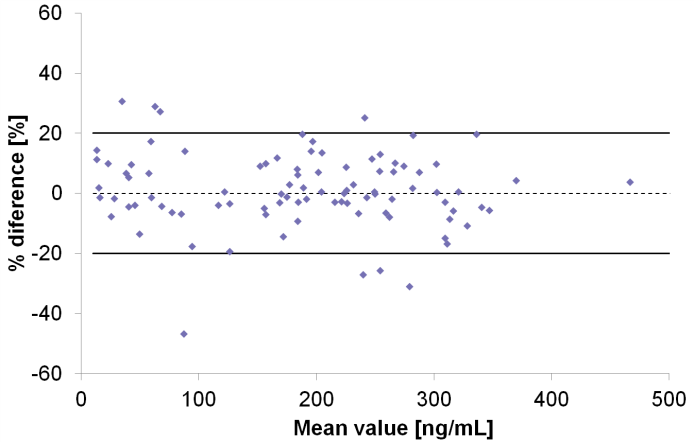 | (B)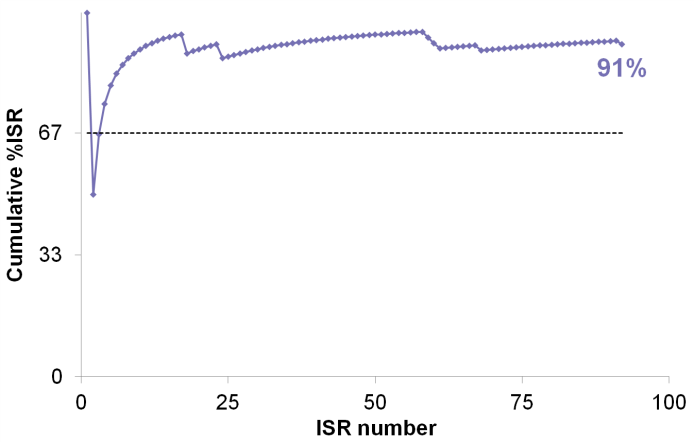 |
| --- | --- |
| (C)  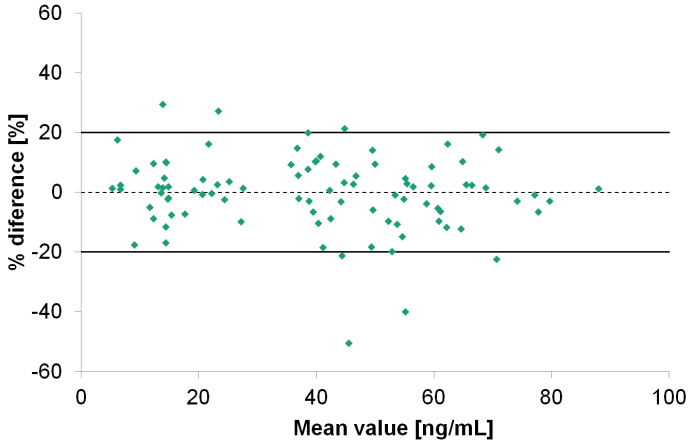 | (D)  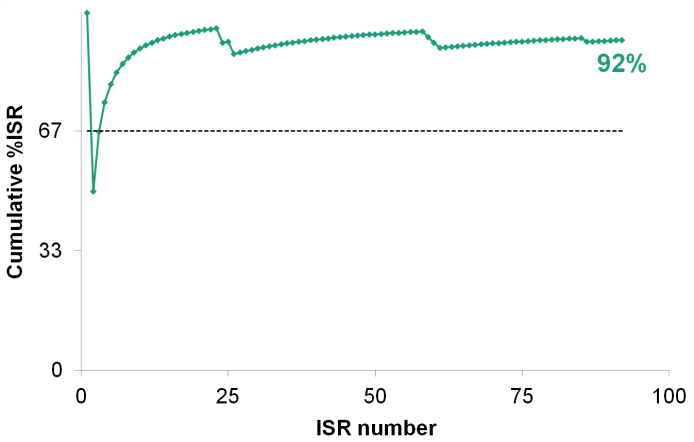 |

Note: colours selected using the qualitative scale and a colour-blind safe option at [colorbrewer2.org](http://colorbrewer2.org/)

**Reference:**

[35] Rudzki PJ, Biecek P, Kaza M. Comprehensive graphical presentation of data from incurred sample reanalysis. Bioanalysis 2017; 9: 947-56.

**Figure S6** Visual presentation of the incurred samples reanalysis (ISR) according to Rudzki et al. [35] in the multiple-dose study for tramadol (violet symbols,
A – %difference versus mean concentration; B – cumulative ISR plot)
and O-desmethyltramadol (green symbols, C – %difference versus mean concentration; D – cumulative ISR plot).

| (A)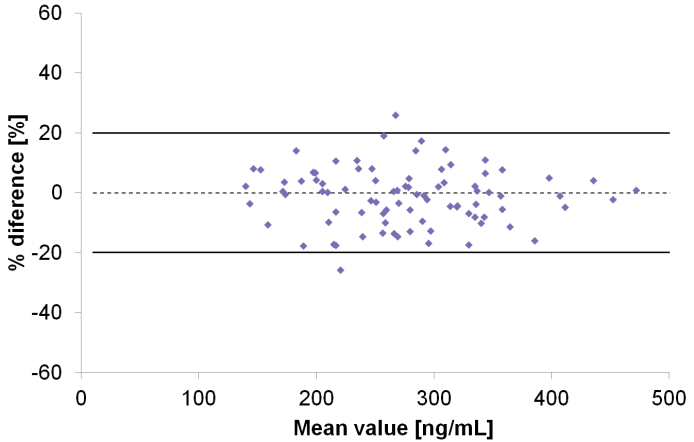 | (B)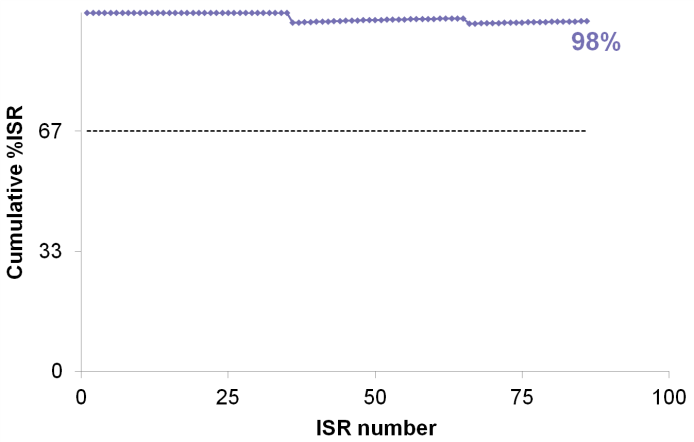 |
| --- | --- |
| (C)  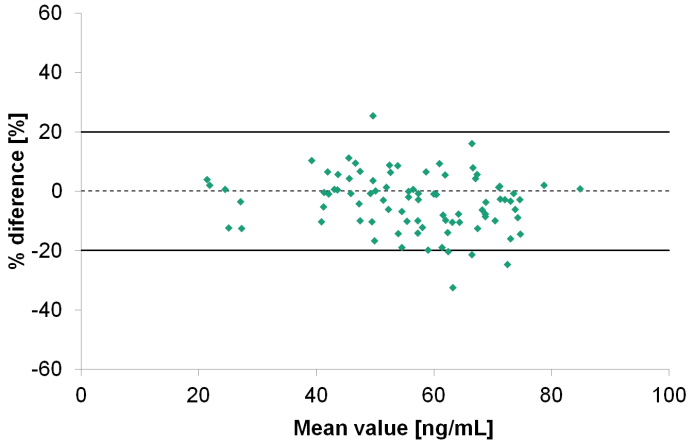 | (D)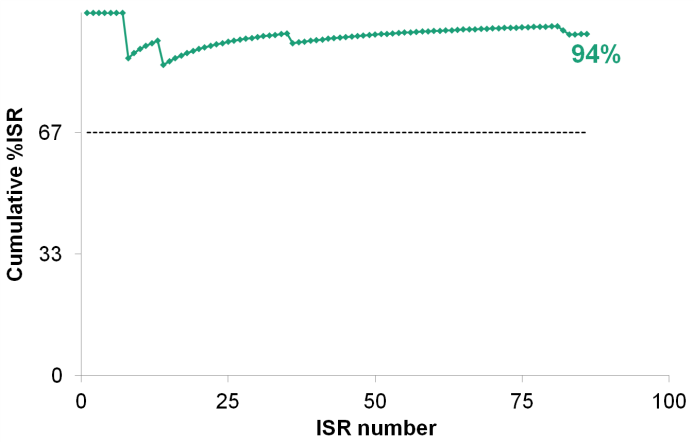 |

Note: colours selected using the qualitative scale and a colour-blind safe option at [colorbrewer2.org](http://colorbrewer2.org/)

**Reference:**

[35] Rudzki PJ, Biecek P, Kaza M. Comprehensive graphical presentation of data from incurred sample reanalysis. Bioanalysis 2017; 9: 947-56.

**Figure S7** Individual tramadol plasma concentration vs. time curves in 25 healthy subjects following a single oral dose of tramadol (2x 50 mg) with magnesium ions (**A**) and tramadol alone (**B**) presented in the linear / linear scale.

**(A) (B)**

**
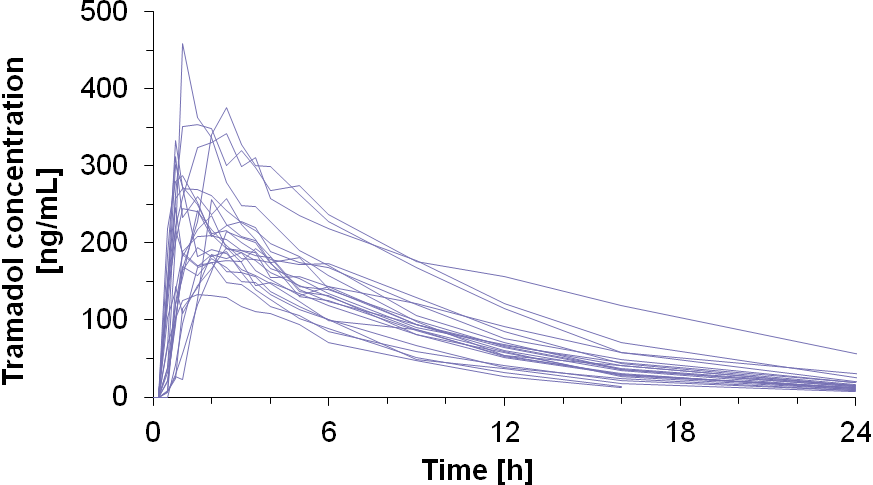

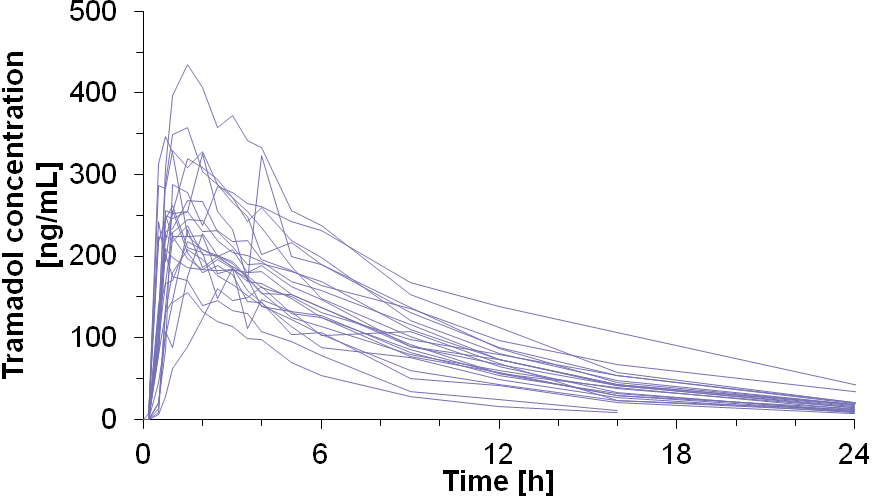
**

**Figure S8** Individual tramadol plasma concentration vs. time curves in 25 healthy subjects following a single oral dose of tramadol (2 x 50 mg) with magnesium ions **(A)** and tramadol alone **(B)** presented in the log / linear scale.

**(A) (B)**


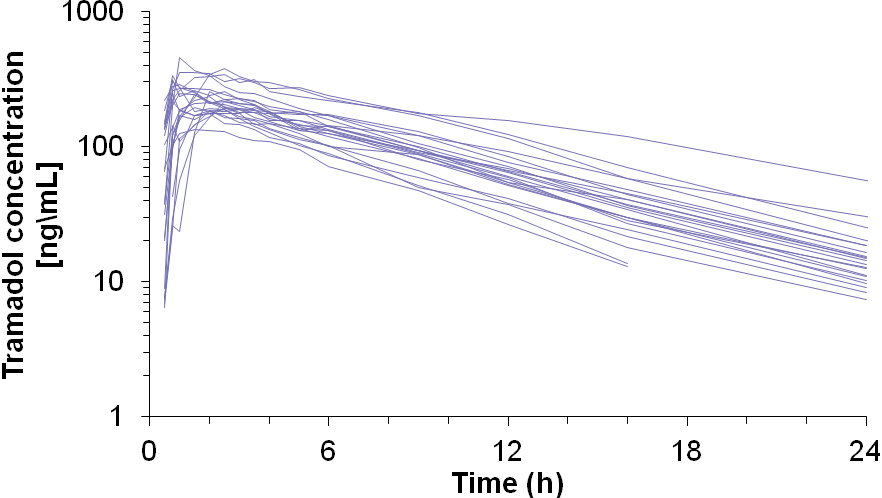

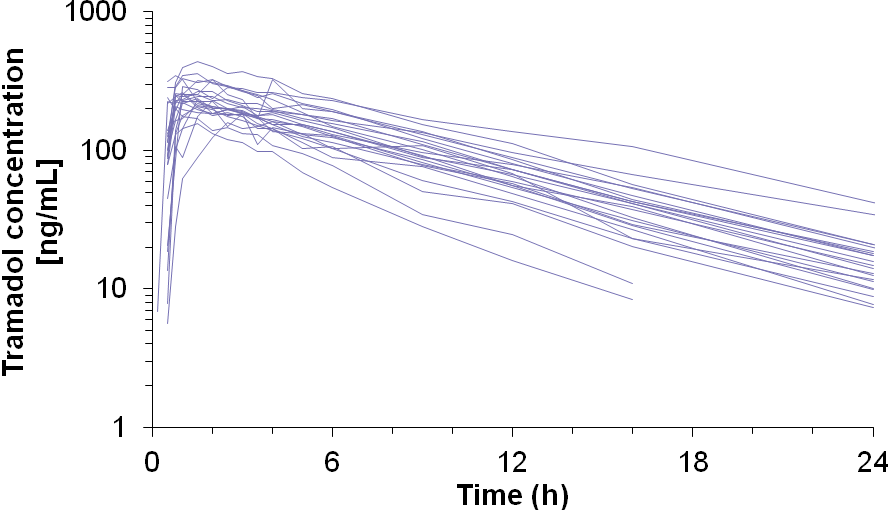


**Figure S9** Individual O-desmethyltramadol plasma concentration vs. time curves in 25 healthy subjects following a single oral dose of tramadol (2x 50 mg) with magnesium ions (**A**) and tramadol alone (**B**) presented in the linear / linear scale.

**(A) (B)**

**
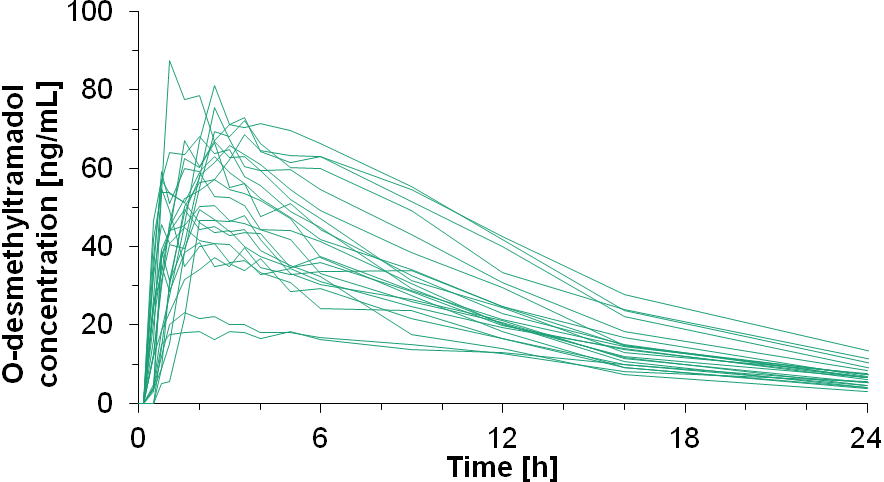

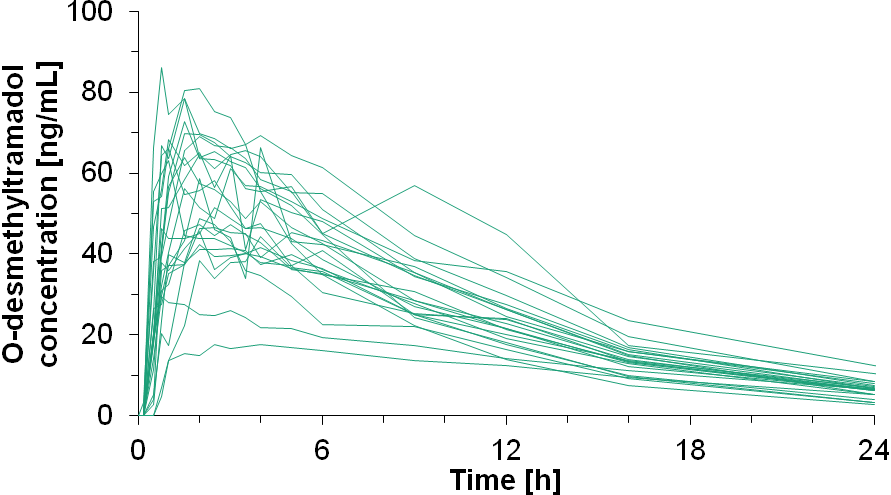
**

**Figure S10** Individual O-desmethyltramadol plasma concentration vs. time curves in 25 healthy subjects following a single oral dose of tramadol (2 x 50 mg) with magnesium ions **(A)** and tramadol alone **(B)** presented in the log / linear scale.

**(A) (B)**


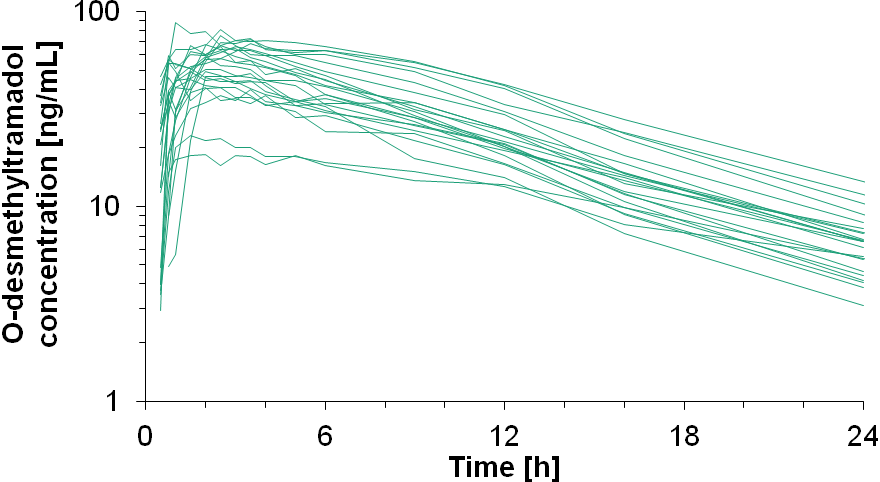

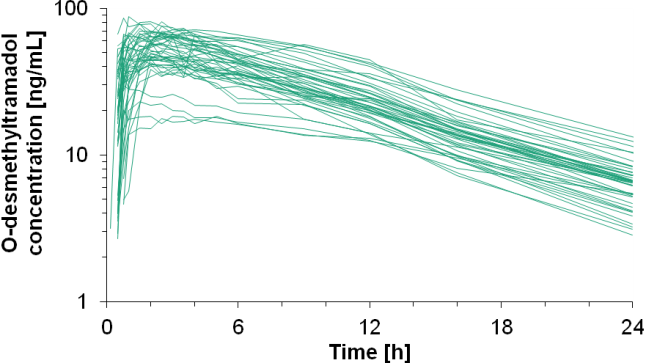


**Figure S11** Individual tramadol plasma concentration vs. time curves in 26 healthy subjects following multiple oral doses of tramadol (50 mg) with magnesium ions (**A**) and tramadol alone (**B**) presented in the linear / linear scale.

**(A) (B)**


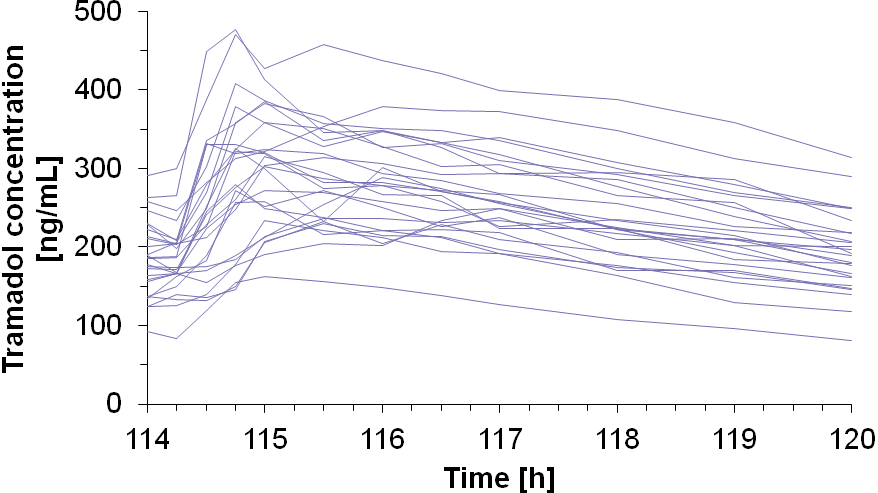

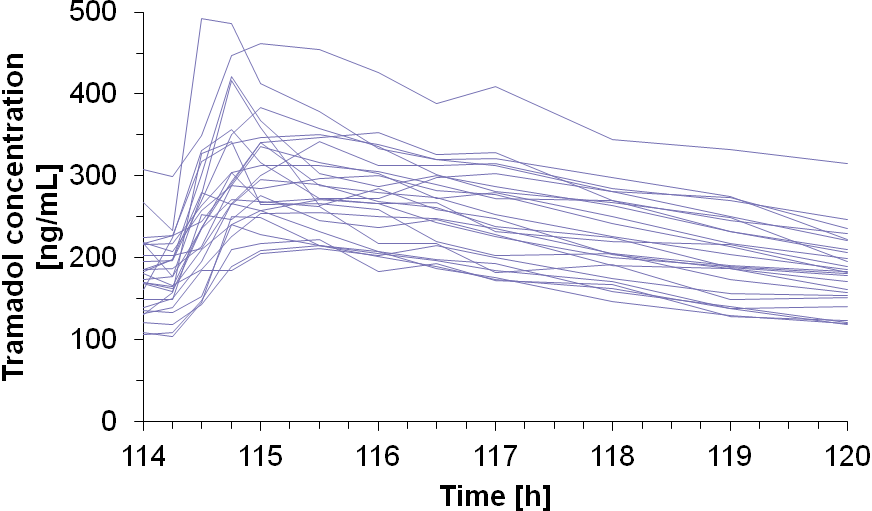


**Figure S12** Individual tramadol plasma concentration vs. time curves in 26 healthy subjects following multiple oral doses of tramadol (50 mg) with magnesium ions **(A)** and tramadol alone **(B)** presented in the log / linear scale.

**(A) (B)**

**
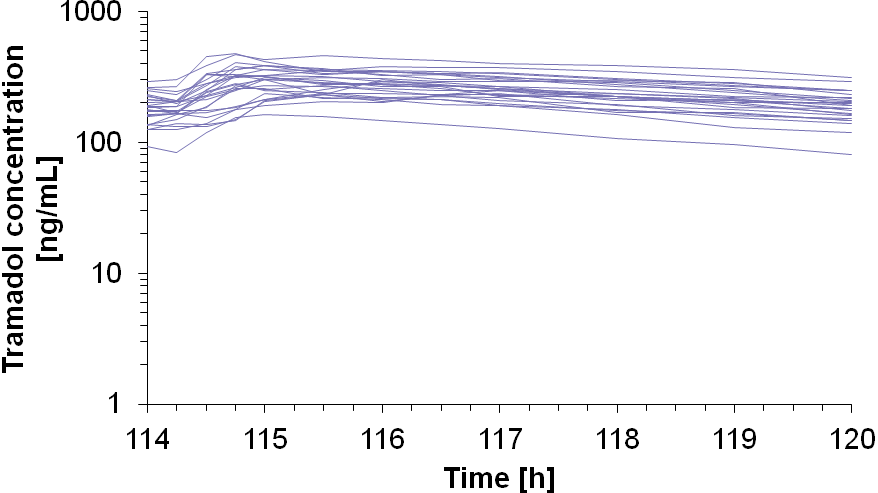

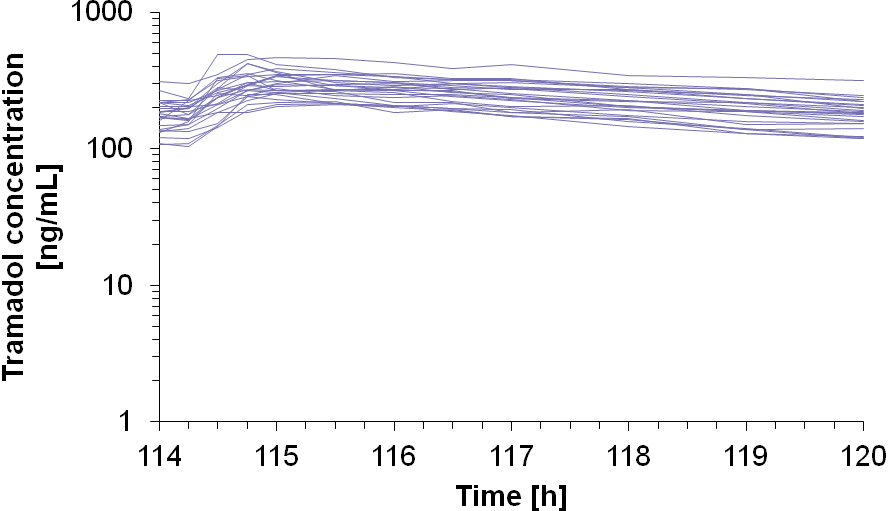
**

**Figure S13** Individual tramadol plasma concentration vs. time curves in 26 healthy subjects following multiple oral doses of tramadol (50 mg) with magnesium ions (**A**) and tramadol alone (**B**) presented in the linear / linear scale.

**(A) (B)**


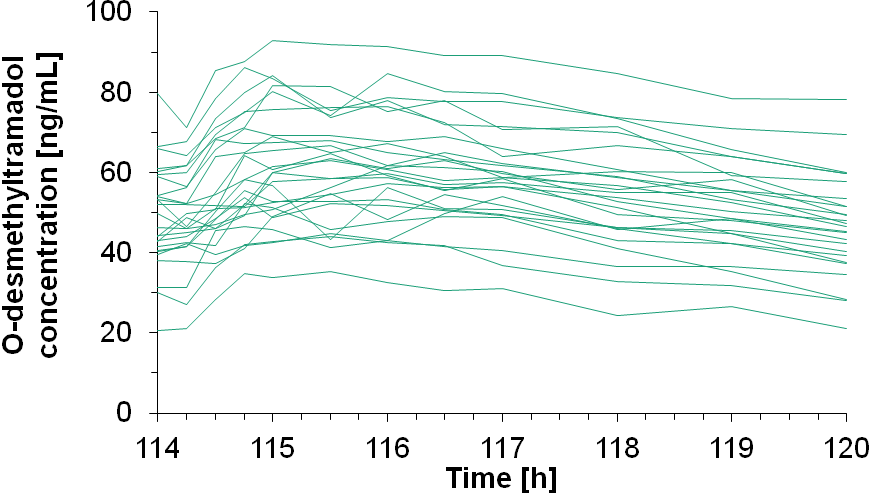

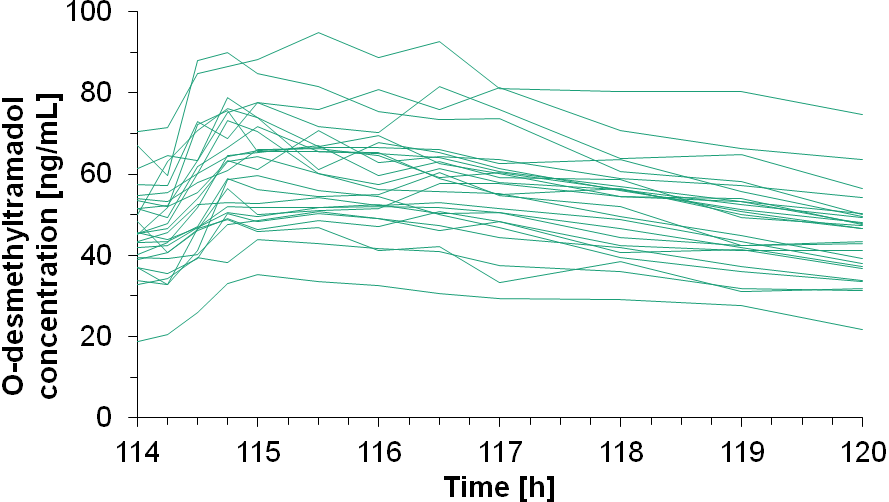


**Figure S14** Individual O-desmethyltramadol plasma concentration vs. time curves in 26 healthy subjects following multiple oral doses of tramadol (50 mg) with magnesium ions **(A)** and tramadol alone **(B)** presented in the log / linear scale.

**(A) (B)**


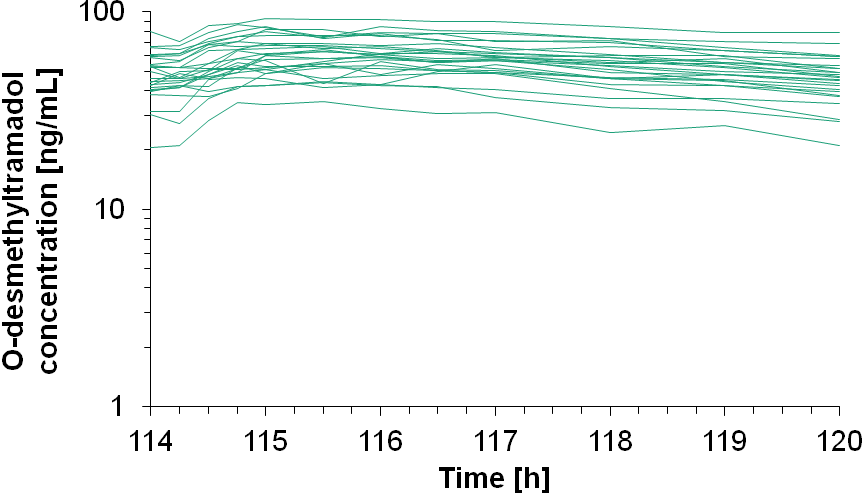

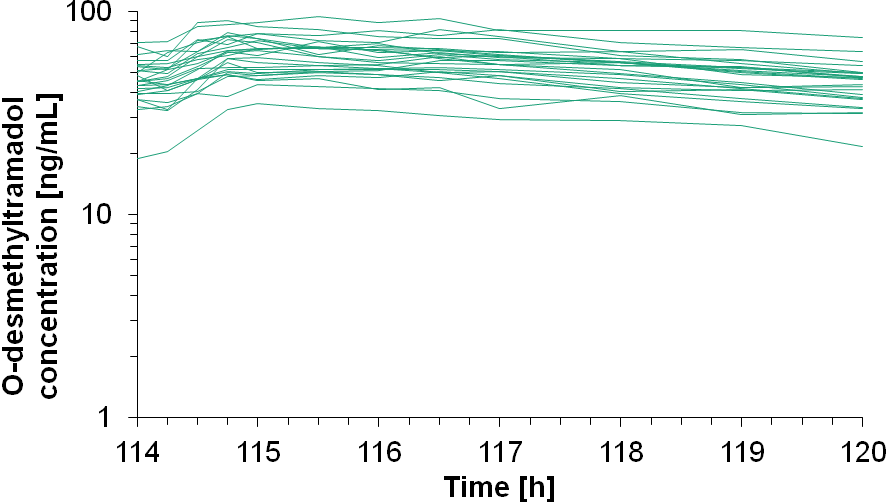

Supplement: Supplementary file 1 — Supplementary file1 (DOCX 1820 KB) [file 43440_2021_239_MOESM1_ESM.docx]
